# Evaluation of an Ipsilateral Uterine Horn Resection and Ovariectomy Surgical Model in Gilts for Embryo Collection

**DOI:** 10.3390/ani15162366

**Published:** 2025-08-12

**Authors:** Mikayla E. Ewasiuk, Richard R. E. Uwiera, Louisa J. Zak, Eli Grindflek, Michael K. Dyck

**Affiliations:** 1 Department of Agricultural, Food, and Nutritional Science, University of Alberta, Edmonton, AB T6G 2P5, Canada; ewasiuk@ualberta.ca; 2 Topigs Norsvin Research Center, Meerendonkweg 25, 5216 TZ Den Bosch, The Netherlands

**Keywords:** embryos, gilts, oocytes, ovariectomy, swine, uterine horn resection surgery

## Abstract

Minimizing the risk of disease transmission, disseminating superior genetics, and reducing transportation costs are recognized advantages of embryo biotechnologies. These advantages make the development of a minimally invasive and repeatable procedure in pigs enticing, but simultaneously magnify the anatomical constraints. For decades, the swine industry has struggled to establish a universal procedure to collect pre-implantation embryos from pigs due to their long and convoluted uterine horns (UHs). Thus, the objectives were to evaluate the benefits of employing a transitional surgical model by shortening UH tissue using a 40 cm ipsilateral resection and assess the compensatory ovulatory response following an ovariectomy. The surgery was deemed successful as the UH was resected and the contralateral UH was fully ligated. The dam- and sire-line gilts exhibited ovarian hypertrophy between surgery and slaughter on the remaining ovary, illustrated by an increase in the number of corpora lutea (13.4 and 3.0 vs. 27.2 and 12; *p* < 0.05, respectively) and intact ovary weight (11.9 and 7.7 vs. 25.9 vs. 38.7 g; *p* < 0.05, respectively). This research is a vital step in assessing whether this interim surgical approach serves as a valuable method to advance the development of non-surgical techniques to collect pre-implantation embryos in pigs.

## Simple Summary

The swine industry has long struggled to develop a reliable and minimally invasive method to collect pre-implantation embryos from pigs due to their complex reproductive anatomy. Embryo biotechnologies offer significant benefits, including reducing disease transmission, improving genetic selection, and lowering transport costs. To address these challenges, a surgical model was tested that shortens the uterine horn using a tissue resection approach and assessed ovarian response following an ovariectomy. The surgery successfully modified the reproductive tract while prompting a compensatory increase in ovarian activity, as observed in higher corpora lutea counts and ovary weights. These findings suggest the approach could potentially advance embryo collection techniques, but further studies are needed to refine the method and ensure it can be applied without surgery. If successful, this research could enhance pig breeding practices, making them more efficient and sustainable, benefiting this agricultural sector and food production.

## 1. Introduction

Recent reports have shown that pork is the second most widely consumed meat product worldwide and is expected to represent 34% of the total animal protein produced in 2030, following poultry at 41% and surpassing beef at 20% [1]. With the growing global population and projected increase in pork consumption, an emphasis on production intensity, specifically the necessity for efficient and environmentally sustainable production practices, must be considered [2,3]. One approach to address these production demands is through the use of embryo biotechnologies and the optimization of herd reproduction metrics, such as ovulation rates and litter size [4,5].

Employing embryo collection and transfer protocols could improve production efficiency by disseminating superior genetics with a minimal risk of disease transmission, reducing transportation costs, and protecting animal welfare [6]. Embryo collection and transfer techniques are commercially and reliably used in the cattle and equine industries; however, their use remains limited in the swine sector and requires further development [7]. A key difference hindering the universal adoption of these reproductive strategies in swine herds is the difficulty manipulating the complex female reproductive anatomy, most notably the rigid cervix and long, coiled uterine horns (UHs) [8].

The two current methods of collecting embryos from pigs are using either surgical techniques or post-slaughter, which have both advantages and disadvantages [8]. The surgical approach requires technically trained personnel as well as specialized facilities to maintain an animal under anesthesia and perform aseptic surgical techniques to access and flush the upper UHs for morula and blastocyst-staged embryos [9]. Despite the technical challenges with this approach, surgical attempts have been rather successful at collecting embryos. Cameron et al. [10] harvested 751 embryos from 37 donors and transferred them into 39 recipient sows, resulting in a nearly 80% farrowing rate with an average litter size of 8.1 ± 3.0 (mean ± sd). The results surrounding post-slaughter embryo collections have also been reported. Wollenberg et al. [11] recovered over 500 porcine embryos post-slaughter from 35 superovulated gilts. Another study found that 85% of porcine embryos exposed to swine vesicular disease fully developed in vitro, which was comparable to the 86% maturation rate of unexposed embryos [12]. This demonstrates the resilience embryos can have against pathogens and highlights the potential to preserve valuable genetics in the event of a disease outbreak [13,14]. However, performing embryo collections post-slaughter is inherently disadvantaged by the fact that it can only be performed once as this is a terminal procedure.

To further improve reproductive efficiency, superovulation strategies can be employed. One study used an exogenous gonadotropin hormone and discovered that sows administered with a dose of 1500 IU had an increased ovulation rate of 45.7 ± 20.3 compared to the pigs that received a lower dose: 28.5 ± 11.7 [15]. Nguyen et al. [16] also exhibited the positive impact of this management technique describing significant differences in the number of follicles (13.56 vs. 7.45), corpora lutea (CL) (12.32 vs. 6.12), and embryos recovered (10.91 vs. 5.67) between superovulated and non-superovulated pigs, respectively. The drawbacks associated with the current strategies prove to be an opportunity to establish a novel embryo collection technique that is repeatable and can assist production herds in attaining their output targets by enhancing reproductive efficiency.

To develop an approach to perform embryo collections in pigs, the first objective of this study was to surgically modify the reproductive anatomy of a gilt by shortening the UH tissue via a 40 cm ipsilateral UH resection to improve accessibility to the utero-tubal junction (UTJ) and pre-implantation embryos. Additionally, a contralateral ovariectomy to the resection site was performed to evaluate the compensatory ovulatory response of a single ovary. The final objective was to validate the success of the surgical procedure post-mortem by evaluating the collection of viable embryos. The corresponding hypothesis predicts that it is possible to successfully modify the reproductive anatomy of a gilt and harvest pre-implantation embryos. The goal is to improve the efficiency and sustainability of pig breeding practices in order to strengthen the agricultural sector and support more resilient food production systems.

## 2. Materials and Methods

### 2.1. General

This study was conducted from August 2022 to October 2023 in accordance with the Canadian Council on Animal Care guidelines and approved by the Institutional Animal Care and Use Committee at the University of Alberta (AUP00003403).

### 2.2. Gilt Selection and Pre-Surgery Cycling

Twenty parity zero (P0) gilts with a mean age of 22.3 ± 2.24 weeks and a mean body weight of 118.5 ± 6.64 kg were selected from two Topigs Norsvin nucleus herds (Plumas and Malonton, MB). These pre-pubertal pigs consisted of two distinct genetic lines: the dam line (*n* = 10) and sire line (*n* = 10). The gilts were transported to the Swine Research and Technology Centre (Edmonton, AB, Canada). Upon arrival, the gilts were quarantined for two weeks in a temperature–humidity-monitored barn (18 ± 2 °C) and housed in pairs of two during acclimation and gradual environmental exposure. During the quarantine period, the gilts were assessed daily for visual signs of the onset of estrus, including vulva reddening, swelling, and mucus discharge. Behavioral estrus signs were also considered, such as the degree of vocalization, ear position, and hind leg stance in response to back pressure.

Following the two-week period, daily estrus detection was performed and assessed by the same visual and behavioral characterizations of estrus during quarantine but while in direct nose-to-nose contact with a boar. To be eligible for surgery, gilts had to exhibit one distinct estrus. Following the gilts’ initial estrus, all subsequent heat checking began on d 18 of the estrous cycle. Surgery was performed on all animals between d 10–15 of their estrous cycle, following the onset of estrus (d 0). At the time of surgery, gilts were 45.71 ± 4.42 weeks old and had a mean body weight of 188.94 ± 7.29 kg.

### 2.3. Surgery

For the procedure, all pigs were fasted from solid food for 8 h prior to surgery. Gilts were premedicated with 2 mg/kg of azaperone (Elanco, Mississauga, ON, Canada) and 2.2 mg/kg of xylazine (Bimeda, Cambridge, ON, Canada), administered intramuscularly (IM). Following mild sedation, an intravenous (IV) ear catheter was inserted. Induction of anesthesia included the administration of 2 mg/kg of ketamine (Vetoquinol, Lavaltrie, QC, Canada) mixed with 1 mg/kg of propofol (Aspen, Oakville, ON, Canada) IV. Pigs were intubated with a cuffed endotracheal tube (11.0 mm inner diameter, 15.7 mm outer diameter; Jorgensen Laboratories Inc., Loveland, CO, USA), maintained on 2–4% isoflurane (Boyle Anaesthetic Machine; MediShield, Rexdale, ON, Canada) at 1890 mL/min O_2_, and ventilated at 15–20 breaths/min (Model 2002; Hallowell EMC, Pittsfield, MA, USA). Following intubation, gilts were positioned in right lateral recumbency. An IM inverted L-line block of 2% lidocaine with epinephrine (Rafter 8, Airdrie, AB, Canada) using a total volume of 30 mL was administered underneath the transverse process of T14-L4 vertebrae and along the caudal aspect of the top third of the distal ribs. The surgical site was prepared by shaving the hair, followed by alternating 4% chlorhexidine (Partnar Animal Health Inc., Ilderton, ON, Canada) scrubs and water washes, and then sprayed with a povidone–iodine solution (10% Betadine, West Penetone, Montreal, QC, Canada). Prior to surgery, the skin surgical site received a final application of 70% isopropyl alcohol (McCarthy Veterinary Supplies, Airdrie, AB, Canada), was draped in a standard four-quadrant pattern, and then covered with an iodine-impregnated disposable adhesive drape (3M Loban 2 Incision Drape, 3M Company, St. Paul, MN, USA). The hemodynamics were preserved with IV fluid therapy (Lactated Ringer’s Solution and 5% dextrose; Baxter, Richmond, BC, Canada) at 5 mL/kg/h for the first hour and then maintained at 2 mL/kg/h for the procedure. Clinical parameters, including heart rate as well as rectal temperature, were maintained throughout the procedure at 71.84 ± 13.10 bpm and 37.25 ± 0.79 °C, respectively.

**Figure 1:**
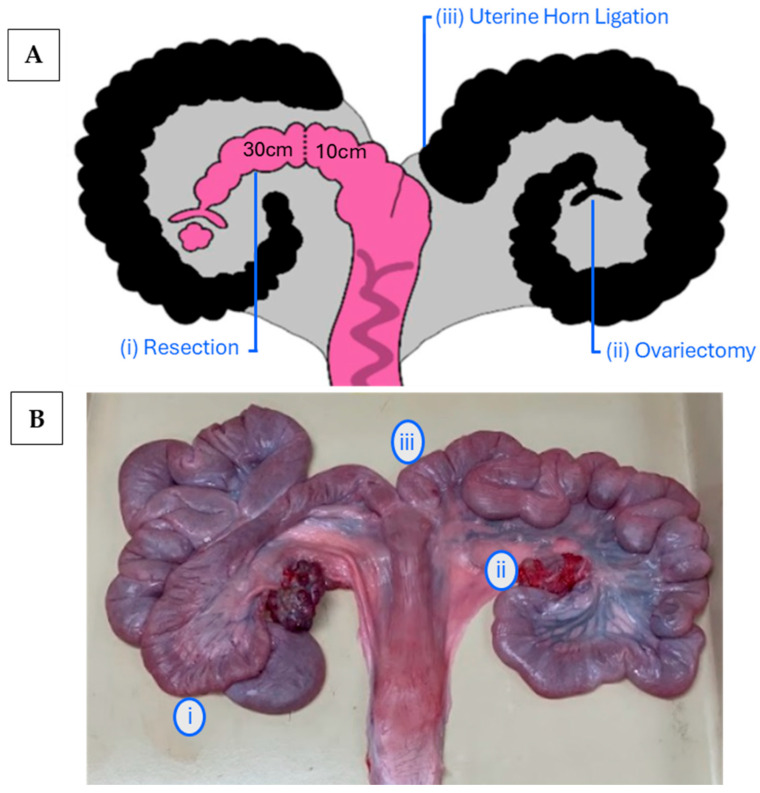
Surgical modification of the gilt reproductive tract performed at d 12.27 ± 1.62 of the estrous cycle. The images illustrate an ipsilateral UH resection (left), contralateral ovariectomy (right), and contralateral UH ligation (right). (**A**) The pink portion represents accessible reproductive tissue for embryo flushing, and black UHs are excluded; the dotted line represents anastomosis; (**B**) healed reproductive tract collected post-mortem.

An approximate 16 cm skin incision within the dorsal aspect of the left paralumbar fossa extending to the ventral abdomen was used to visualize the underlying muscle. The muscle layers were separated with either sharp or blunt dissection, and the peritoneum was opened, allowing for the exteriorization of the UHs. Modification of the uterus was used to create a ‘single-sided’ (i.e., ipsilateral) UH surgical model for embryo collection. The three-stage procedure is illustrated in Figure 1 and included the following steps: (i) Ipsilateral resected UH for embryo collection. Surgical procedure: A 40 cm segment of the UH was resected by transecting the horn 30 cm distal of the UTJ and 10 cm proximal from the bifurcation of the uterine body. Broad ligament vessels were ligated with 2-0 polyglactin suture (Vicryl, Ethicon, Raritan, NJ, USA), and a simple continuous pattern with a 2-0 polyglactin suture was used to anastomose the cranial and caudal segments of the ipsilateral UH. (ii) Contralateral ovariectomy was used to prevent ovulation into the contralateral horn. Surgical procedure: The ovarian pedicle was crushed in two positions, and the crushed tissue closest to the ovary was transfixed and double-ligatured with 1 polyglactin suture (Vicryl, Ethicon, Raritan, NJ, USA), ensuring complete homeostasis. The ovary was carefully excised from the ligated ovarian pedicle. (iii) Contralateral UH ligation to ensure proper catheter insertion into the ipsilateral horn by ligating close to the distal aspect of the contralateral horn. Surgical procedure: At the cranial segment of the uterine body bifurcation, the contralateral horn was transected and then both the caudal end of the contralateral UH and cranial end of the uterine body were closed with a Parker–Kerr suture pattern using a 2-0 polyglactin suture. Following the surgery, the UHs were administered with 20 mg/kg of cefazolin (Fresenius Kabi, Toronto, ON, Canada) and the uterus was replaced into the abdomen. The individual muscle layers were closed with a simple continuous suture pattern with 2-0 polydioxanone (PDS II Ethicon, Raritan, NJ, USA) and the skin closed with a subcuticular suture pattern using a 2-0 polyglactin suture. For prophylactic antibiotic therapy and post-surgical analgesia, 5 mg/kg of ceftiofur (Zoetis, Kirkland, QC, Canada) and 0.4 mg/kg of metacam (Boehringer Ingelheim, Burlington, ON, Canada) were administered IM and 0.01–0.02 mg/kg of slow-release buprenorphine (Chiron, Guelph, ON, Canada) was administered subcutaneously. In addition, 24 h post-surgery, all gilts received 0.4 mg/kg of meloxicam per os over a four-day period (Solvet, Calgary, AB, Canada).

### 2.4. Ovary Dissection—Contralateral Ovariectomy

For the excised ovary, the number of follicles and CLs were counted and measurements for total intact ovary weight and displacement were recorded. Ovary displacement was evaluated by placing the intact ovary in a water-filled graduated cylinder and calculating the difference in water position.

### 2.5. Animal Husbandry and Post-Surgical Management

All gilts were fed twice daily at 700 h and 1400 h and had ad libitum access to water. Gilts were housed individually in farrowing crates for one week following surgery to limit movement and for ease of recovery. During the recovery period, individual gilts were assessed daily for general health, paying particular attention to surgical wound healing and indicators of surgical failure with infection, namely elevated body temperature, inappetence, depression, and lethargy. Visual estrus detection was conducted from d 18 of the estrous cycle until seven days post-surgery, after which a boar was re-introduced while performing estrus detection. Gilts that exhibited three normal estrous cycles following the surgical procedure were considered to have fully recovered.

### 2.6. Collection and Assessment of Reproductive Tissues

Gilts’ estrous cycles were synchronized using altrenogest (Regumate, Merck Animal Health, Kenilworth, NJ, USA) from 10 to 12 days (9 mL/gilt/day) prior to euthanasia. The gilts were artificially inseminated on their first estrus following altrenogest withdrawal with an interval of 24 h until the end of standing heat (0 h, 24 h, 48 h). Following breeding, gilts were euthanized via captive bolt and exsanguination at d 4–7 of the estrous cycle. At the time of euthanasia, the gilts were 81.14 ± 6.43 weeks old and had a mean body weight of 234.25 ± 8.10 kg. The reproductive tract was recovered from each gilt immediately following euthanasia, and embryo collection was conducted. Following the removal of the broad ligament, the resected UH was clamped at the bifurcation. The resected UH was flushed with 50 °C pre-warmed embryo media and 30 mL aliquots were injected into the uterine lumen with a blunt needle syringe inserted through the UH at the UTJ. A modified Tyrode’s lactate (TL)–HEPES (H)–polyvinyl alcohol (PVA) embryo media was used [17]. Adjustments to the TL–H–PVA medium included a reverse osmosis water base containing potassium penicillin G (65 µg/mL) and monosodium phosphate (0.34 mM) instead of monopotassium phosphate (Sigma-Aldrich; MilliporeSigma Canada Ltd., Oakville, ON, Canada). The final media pH (Thermo Fisher Scientific, Edmonton, AB, Canada) and osmolarity were adjusted to 7.4 and 290 mOsm/kg by incrementally adding sodium hydroxide (1 M). The final TL–H–PVA media was sterilely vacuum filtered (0.22 µm) and stored from 4 to 6 °C for a maximum of two weeks. The resected UH was flushed four to five times in an attempt to harvest the majority of oocytes and embryos present. The embryo media of each sequential flush were collected in separate 100 mL vials through a curved glass funnel at the bifurcation. The flush media were transferred into Petri dishes and evaluated for oocytes and pre-implantation embryos using a stereomicroscope (LEICA MZ125, Leica Microsystems, Wetzlar, Germany). Each pre-implantation embryo was assessed individually and recorded for its relative stage of development and viability based on subjective visual categorization, illustrated in Table 1. The developmental stage was classified as an oocyte, 2–4 cell, 4–8 cell, or morula, and was determined by the number of cells inside the zona pellucida and the degree of compaction. Oocytes and embryos were further categorized as normal or degenerative based on size, morphology of the zona pellucida, and the spherical nature of the inner embryonic cells. Percent embryo recovery was calculated by dividing the number of recovered oocytes and embryos by the number of fresh ovulations of follicles observed on the ovary. Measurements were recorded for all segments of the modified UH, contralateral intact UH, uterine body, cervix, cranial and caudal vagina, as well as the ipsilateral and contralateral oviducts relative to the resection. Ovary parameters were measured using the same protocol as described earlier (Section 2.4. Ovary Dissection—Contralateral Ovariectomy).

### 2.7. Statistical Analysis

The experimental unit was each individual gilt. Reference to “embryos” includes both developing embryos and potential embryos (i.e., oocytes). All statistical analyses were computed using the SAS Software, Version 9.4 (SAS Institute Inc., Cary, NC, USA). A t-test was performed to compare the length of the estrous cycle pre- and post-surgery. For the ovary data, normality was verified using a Shapiro–Wilk test to assess model assumptions, and the best-fit distribution was used in PROC GLM. Tukey adjustments were made for multiple comparisons, including pairwise comparisons between breeds, time points, and between the breed x time interactions. Statistical significance was declared at a *p*-value of ≤0.05. The statistical model used for the ovary data is as follows:

(1) $$Y\;=\;\mu \;+\;B_{i}\;+\;T_{j}+B_{i}\times T_{j}+\;\varepsilon_{ijk},$$

where μ is the sample mean, $B_{i}$ is the effect of breed, $T_{j}$ is the effect of time, $B_{i}\times T_{j}$ is the effect of breed × time interaction, and $\varepsilon_{ijk\;}$ is the experimental error term. The model fit was tested by the Bayesian information criterion (BIC) and the model with the lowest BIC was used. To account for any unbalanced effect, Kenward-Roger2 was used to estimate the degrees of freedom. The least squares mean (LSM), standard deviation (SD), and standard error of the mean (SEM) were reported for data comparisons. Outlier analysis was performed, and if identified, was removed when exceeding three standard deviations from the mean.

## 3. Results

Five gilts were removed from this study due to either lameness with poor lower limb conformation or procedural failure. Of the gilts that underwent surgery (*n* = 15), select gilts were excluded from this study and the statistical analyses for reproductive reasons (*n* = 4) and post-surgical health concerns (i.e., lower limb lameness, *n* = 3). Therefore, the statistical analyses were performed on eight gilts (*n* = 8); surgery was performed at d 12.27 ± 1.62 of the estrous cycle and d 5.36 ± 0.92 at the time of euthanasia.

### 3.1. Estrous Cyclicity

Prior to surgery, the gilts underwent 3 to 6 estrous cycles with a mean cycle length of 20.89 ± 0.66 d. Following surgery and prior to slaughter, the gilts cycled between 8 and 13 times, with an average interval of 21.53 ± 0.68 days. The t-test analysis revealed that the length of the estrous cycle was statistically different between time points, pre- and post-surgery (*p* < 0.01), indicating that the estrous cycle was prolonged following surgical manipulation of the gilt reproductive tract. The estrous cycles in which altrenogest was administered were excluded.

### 3.2. Reproductive Tract Measurements

The mean length of the intact, ligated UH was 97.00 ± 15.98 cm. The ipsilateral resection was 36.63 ± 3.49 cm; 23.79 ± 4.14 cm from the UTJ to the anastomosis and 12.64 ± 2.39 cm from the anastomosis to the bifurcation of the uterine body. The mean lengths of the uterine body and cervix were 6.56 ± 2.09 cm and 18.31 ± 5.04 cm, respectively. The vagina was differentiated into the cranial (19.63 ± 5.01 cm) and caudal (12.44 ± 0.94 cm) segments and had a total mean length of 32.06 ± 5.05 cm. The oviduct ipsilateral to the resection was 28.50 ± 0.82 cm, while the contralateral oviduct was 27.88 ± 1.69 cm. The reproductive tract measurements, including the means, standard deviations, and minimum and maximum lengths, are summarized in Table 2.

### 3.3. Ovary Parameters—Count Data

**Figure 2:**
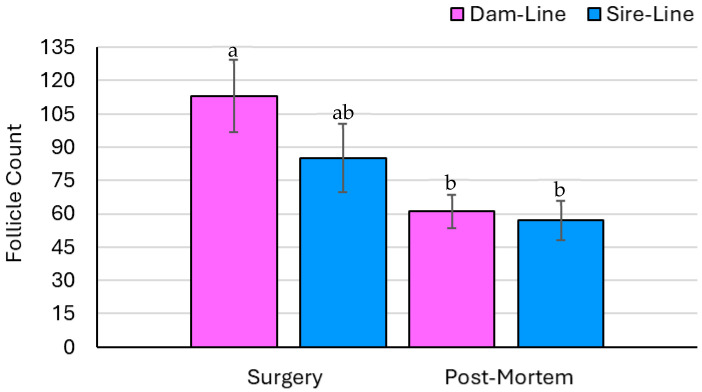
The number of ovarian follicles collected from the gilts (*n* = 8) was differentiated by breed (dam and sire genetic lines) and the time of recovery (surgery and post-mortem). Significant findings (*p* < 0.05) are indicated by different letters. There was a significant difference between time points (*p* < 0.05), but no effect of breed (*p* = 0.20) or the breed × time interaction (*p* = 0.95). The number of follicles decreased from surgery to the non-surgical procedure (i.e., post-mortem euthanasia) in the dam-line gilts and was not statistically different between time points in the sire-line gilts.

**Figure 3:**
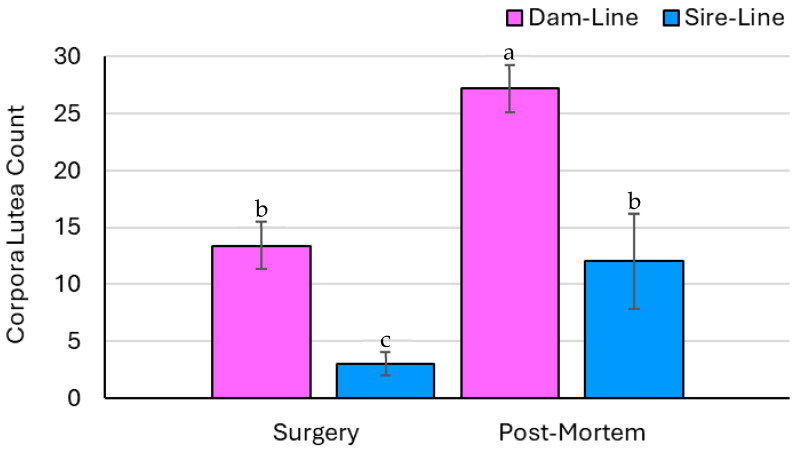
The number of ovarian CLs collected from the gilts (*n* = 8) was differentiated by breed (dam and sire genetic lines) and the time of recovery (surgery and post-mortem). Significant findings (*p* < 0.05) are indicated by different letters. A breed and time effect was observed (*p* < 0.05), while the breed × time interaction was statistically insignificant (*p* = 0.77). The number of CLs significantly increased from surgery to the non-surgical procedure (i.e., post-mortem euthanasia) in both breeds.

The number of follicles was not affected by breed or the breed × time interaction but was affected by time (*p* = 0.20, *p* = 0.95, and *p* < 0.05, respectively; Figure 2). The overall number of ovarian follicles significantly decreased from the surgical ovariectomy to the collection of the remaining ovary post-mortem. The number of CLs was affected by breed and time but was not affected by the breed × time interaction (*p* < 0.01, *p* < 0.01, and *p* = 0.17, respectively; Figure 3). Specifically, the number of CLs increased on the ovary recovered following euthanasia compared to the excised ovary collected during surgery in both the dam- and sire-line breeds. Out of the eight gilts, five had ovarian cysts, ranging between one and seven per animal.

### 3.4. Ovary Parameters—Measured Data

**Figure 4:**
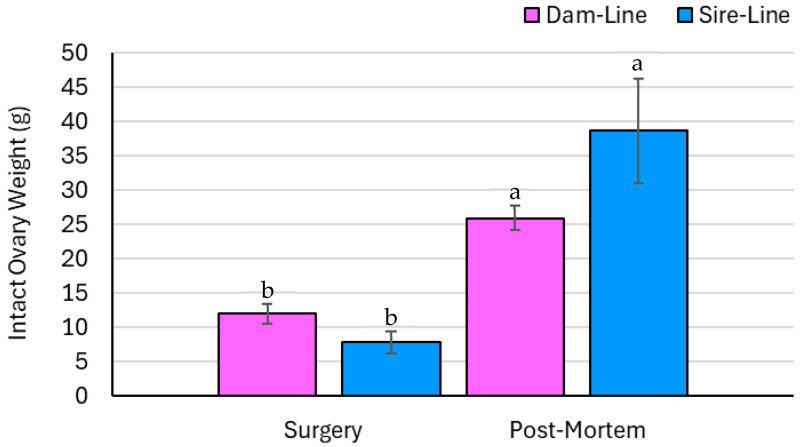
A breed × time interaction (*p* < 0.05) and time (*p* < 0.01) effect was observed in the weight (g) of intact ovaries collected from gilts (*n* = 8), with measurements differentiated by dam and sire genetic lines and by recovery time (surgery and post-mortem). Significant findings (*p* < 0.05) are indicated by different letters. The effect of breed was not statistically significant (*p* = 0.77). Ovarian weight increased over time from surgery to euthanasia in both dam- and sire-line gilts. There was no significant difference between breeds.

**Figure 5:**
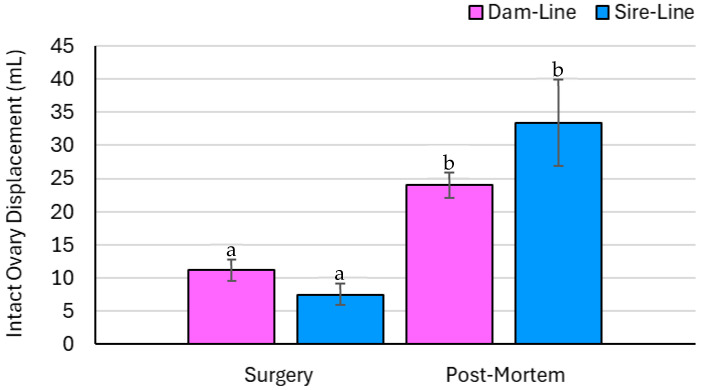
A breed × time interaction (*p* < 0.05) and time (*p* < 0.01) effect was observed in the ovarian displacement (mL) of intact ovaries collected from gilts (*n* = 8), with values analyzed across dam and sire genetic lines and recovery time points (surgery and post-mortem). Significant findings (*p* < 0.05) are indicated by different letters. The effect of breed was not statistically significant (*p* = 0.76). Ovarian displacement increased over time from surgery to euthanasia in both dam- and sire-line gilts. There was no significant difference between breeds.

Intact ovary weight was not affected by breed but was affected by time and the breed × time interaction (*p* = 0.77, *p* < 0.01, and *p* < 0.05, respectively; Figure 4). The ovary recovered post-mortem in both the sire- and dam-line gilts had a greater total weight compared to the ovary excised during surgery. Intact ovary displacement was not affected by breed, but statistical significance was detected by time and the breed × time interaction (*p* = 0.76, *p* < 0.01, and *p* < 0.05, respectively; Figure 5). Ovary displacement was greater on the ovary recovered post-mortem for dam- and sire-line gilts.

### 3.5. Post-Slaughter Embryo Recovery

Across the eight post-slaughter embryo collections performed, the embryo medium recovery was 94.0 ± 2.1%. A total of 90 oocytes and embryos were recovered: 18 unfertilized oocytes, 4 fertilized oocytes, 1 two to four cells, 37 four to eight cells, and 30 morulae, as seen in Table 3. The overall percent embryo recovery was 56.6 ± 16.3%, with a range of 0 to 30 oocytes and embryos collected per gilt.

## 4. Discussion

### 4.1. Ovulatory Compensation from Surgery to Slaughter

The number of follicles at the time of surgery in the dam-line gilts was comparable to a previous study that assessed six-month-old gilts between d 10 and 14 of the estrous cycle (119 vs. 100, respectively) [18]. The sire-line gilts had fewer ovarian follicles relative to the dam-line gilts, which may be associated with their genetic lineage and selection for increased prolificacy in the dam lines [19]. The selection pressure in sire-line pigs predominantly focuses on growth and carcass traits, such as average daily gain and feed efficiency, with reduced prominence on reproductive traits [20]. Between surgery and slaughter, the number of ovarian follicles decreased in both the dam- and sire-line pigs but was only significantly different in the dam-line gilts between time points, as seen in Figure 2. At the time of surgery, the excised ovary was harvested during the late luteal phase, whereas at slaughter, the ovary was collected early in the luteal phase, which could account for the difference in the number of follicles [21]. In general, following estrus and ovulation, the number of follicles is lower relative to the later stages in the luteal phase [22]. The small sample size and uneven distribution of animals per genetic line could account for the lack of difference in the number of follicles between ovaries in the sire-line gilts. In both the dam- and sire-line gilts, the number of CLs more than doubled between surgery and euthanasia, visualized in Figure 3. Such findings could indicate that the remaining ovary underwent ovarian hypertrophy following the unilateral ovariectomy, a biological process in which a higher number of follicles are recruited and selected to become dominant and ovulate [21]. Therefore, the CLs on the remaining ovary may have compensated for the loss of the CLs on the excised ovary during surgery [23].

The intact ovary weights significantly differed between time points, markedly increasing from surgery to slaughter in both the dam- and sire-line pigs, illustrated in Figure 4. The mean intact ovary weight of the dam-line gilts at the time of surgery was comparable to sow ovary weights reported by Małopolska et al. [24] of approximately 10.0 g per ovary. Dam-line pigs are genetically selected with a focus on reproductive performance traits, including age at puberty, ovulation rate, embryo survival, litter weight, and wean to estrus interval [25,26]. Therefore, it was expected that the mean ovarian weight in dam-line gilts would be higher than that of sire-line gilts. As a result of the marked increase in the number of CLs recorded on the ovary at slaughter, it is reasonable that an equivalent increase was observed in the intact ovary weight for both breeds. As described earlier, this increase in weight could be explained by the ovarian hypertrophy that occurred, particularly in the increased number of CLs following the ovariectomy [27]. Significantly higher water displacement (i.e., ovary volume) was detected in the post-mortem ovaries relative to those collected at the time of surgery. Although previous studies have not evaluated this parameter, the trend aligns with intact ovary weight, likely because the ovary was not altered between these measurements.

### 4.2. Physiological Outcomes of Ipsilateral Uterine Horn Resection Surgery

The ipsilateral UH resection was intended to be 40 cm across all gilts, although the measured lengths varied between 32.0 and 41.0 cm post-mortem. In a preceding study with similar objectives, a bilateral UH resection surgery was performed in sows, and variations in resection length were also observed, ranging between 7.6 and 25.5 cm in comparison to the intended 17 cm [28]. The cause for these differences between the intended and modified uterine lengths is likely associated with the degree of post-operative shrinkage following separation from the body [29]. The literature regarding how pig tissues respond following surgical manipulation is sparse. However, studies on humans have reported sizeable proportions of tissue shrinkage between surgery and pathological reassessment. After 20 min, Goldstein et al. [30] found a 40% reduction in the length of a resected colon between in vivo and in vitro measurements. In the present study, the time interval between death (i.e., post-slaughter euthanasia) and the removal of the reproductive tract from the body cavity ranged between 20 and 30 min, during which time the post-euthanasia contraction of the uterine tissue could have occurred [31]. Therefore, the difference between the 40 cm resection measurements taken during surgery compared to the shorter lengths measured following euthanasia could have been affected by this time lapse. Despite the differences between humans and pigs, it is possible that there is some physiological overlap regarding how tissue contracts over time. Lastly, the introduction of measurement error could have ensued while evaluating the length of the resection. The precise landmarking of the resection from the bifurcation to the oviduct was variable. This was because of the minor differences in the surgical ligation of the contralateral UH due to the technical challenges of the surgical procedure and the changes in the uterine body following the healing process. However, in all eight gilts, the contralateral UH was successfully ligated from the resection site. Regardless of these anatomical differences in the resection length of the uterus, the impact on the experimental design was likely insignificant and did not impede the ability to collect embryos post-slaughter.

All gilts retained their estrous cycles following the ipsilateral UH resection, but the duration of the estrous cycle was longer following the surgical procedure. Although there was a significant difference between time points, the mean estrous length of 21.53 ± 0.68 d remained within the normal range of a pig’s estrous cycle, which typically ranges from 18 to 24 d [32]. The prolonged estrous cycle length could have been influenced by the presence of ovarian cysts on the remaining ovary, which varied between one and seven per gilt [33]. However, a single ovarian cyst rarely induces adverse effects on the estrous cycle or the fertility of pigs [34]. The present study’s results aligned with this observation, as the gilts with a single cyst had an estrous cycle of similar length pre- and post-surgery or had a relatively high number of embryos recovered post-slaughter. In contrast, the presence of several large cysts can secrete elevated levels of estrogen, resulting in prolonged estrous cycles or the failure to conceive despite repeated inseminations [34]. In this study, some of the gilts with the highest numbers of ovarian cysts had greater differences in estrous length pre- and post-surgery and fewer embryos recovered post-slaughter. In contrast to our investigation, Hazeleger et al. [35] did not detect changes in the length of the estrous cycle in sows that underwent a bilateral UH resection without an ovariectomy.

Alternatively, the prolonged estrous cycle length may have been associated with the higher number of CLs requiring an extended interval for luteolysis to take place. Luteolysis is the physiological process in which prostaglandin F2alpha (PGF2α) is endogenously secreted through countercurrent exchange between the uterine tissue and the ipsilateral ovary, around d 15–16 of the estrous cycle to permit the regression of CLs to corpus albicans [21,36]. Due to the ovarian hypertrophy observed in this study, the greater number of CLs on the single remaining ovary may have taken longer to regress and hence prolonged the length of the gilt’s estrous cycle following surgery. As mentioned earlier, a study that performed a bilateral UH resection did not detect a difference in estrous cycle length pre- and post-surgery; however, the sows in this study also had 10 fewer CLs than the dam-line gilts in the present study [35,37]. If similar estrous cycle changes are observed in the future, this could be addressed by administering an exogenous form of PGF2α after d 13 of the estrous cycle when the affinity for the prostaglandin markedly increases [38]. Collectively, the presence of both endogenous and exogenous prostaglandins may compound to assist the regression of a higher proportion of CLs within the expected time period and result in a normal estrous cycle length. It appears there were physiological changes associated with the removal of one ovary; however, further research is required to determine whether these changes were associated with the development of ovarian cysts or an extended luteolysis period following superovulation.

### 4.3. Post-Slaughter Embryo Collection

Harvesting pre-implantation embryos post-slaughter has been proven to be an effective method for retrieving embryos from pigs [11,39]. In the present study, the mean embryo media recovery was close to 95%, and 90 oocytes and embryos were retrieved. Despite the high volumes of media recovered, there was a large range in the number of oocytes and embryos collected per gilt, ranging between 0 and 30. It is reasonable to assume that a higher embryo media recovery rate is the result of completely flushing the area in which embryos reside along with their retrieval. Thus, the procedures used to flush the ipsilateral UH were likely efficient and collected the majority, if not all, of the oocytes and embryos present within the horn.

Upon the retrieval of embryo media from the gilts in which no or few oocytes and embryos were collected, it was observed that the color of the media was slightly yellow. This change in the qualitative property could have been indicative of the presence of transudate associated with endometritis following surgical manipulation or other serous fluid secreted by the endometrium. Endometritis was also observed in select sows following bilateral UH resection surgery [35]. As a result, the mean embryo recovery of approximately 57% was lower than what has previously been reported in other swine embryo collection studies, averaging between 79 and 84% [11,40]. Therefore, the discrepancy in the number of oocytes and embryos harvested and the number of ovulations may have been a consequence of an unfavorable uterine environment for embryonic development, leading to embryo degeneration prior to the collection [41]. A method to determine the suitability of the uterine environment for embryonic development following uterine surgery could be to measure the pH of the uterus in situ prior to attempting an embryo collection [42]. López-Albors et al. [43] observed that the mean pH of a pig uterus following artificial insemination was around 7.1. Similarly, Nichol et al. [44] measured the pH near the oviduct at different time points throughout the estrous cycle. Near the ampulla–isthmic junction, the pH averaged between 7.2 and 7.3 following ovulation and was more stable likely due to early developing embryos requiring a more constant environment. Previous studies have illustrated that the presence of bacteria can disrupt the homeostatic pH of the uterine environment by increasing it above 8 [45]. In addition, the inflammatory process associated with surgery and post-surgical healing without bacterial contamination can also alter pH [46,47,48]. Utilizing a quantitative measurement, such as pH, may help to explain high media recovery rates and low embryo counts in future swine embryo research if discrepancies are consistently observed.

## 5. Conclusions

This study successfully achieved its objectives by surgically modifying the reproductive anatomy of a gilt through ipsilateral UH resection, enhancing access to the UTJ and pre-implantation embryos. The compensatory ovulatory response of a single ovary was effectively evaluated via contralateral ovariectomy, and a post-mortem assessment confirmed the procedure’s success through the collection of viable pre-implantation embryos, supporting the initial hypothesis.

The surgical procedure employed to alter the reproductive tract of gilts through an ipsilateral UH resection was effective. The procedure successfully shortened the UH, isolated the contralateral horn, and, to an extent, supported early embryonic development. However, it must be noted that the use of an invasive procedure can lead to inadvertent complications, such as uterine contamination, uterine fibrosis, and strictures, which may negatively affect early embryonic survival rates. As such, these complications must be considered prior to the application of this technique for embryo collection. On the other hand, the value of performing an ovariectomy is less conclusive. While superovulation has the potential to improve reproductive efficiency and a greater number of ovulated follicles, this response did not consistently yield a significantly higher recovery of oocytes and embryos across all gilts. Furthermore, the ovarian hypertrophy observed post-ovariectomy appeared to predispose the gilts to ovarian cyst formation and may have extended the amount of time required for the CLs to regress, thereby prolonging the duration of their estrous cycles. Despite the limitations encountered in this study, as a research model, this technique could serve as a key method to develop a repeatable embryo collection procedure by reducing the anatomical barriers that have hampered the commercial application of utilizing swine embryo collections for decades.

## Figures and Tables

**Table 1:** Pre-implantation oocyte and embryo classification based on the stages of development collected during post-slaughter embryo collections.

Unfertilized Oocyte	Fertilized Oocyte	2-Cell	4-Cell	8-Cell	Morula
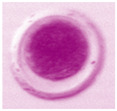	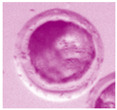	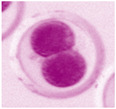	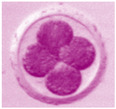	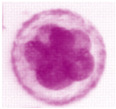	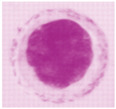

**Table 2:** The reproductive tract measurements recorded post-mortem (*n* = 8) included as means, standard deviations (SDs), and maximum and minimum lengths. The euthanasia of pigs was performed following estrus and artificial insemination in the early luteal phase of the estrous cycle (d 5.36 ± 0.92).

Measured (cm)	Mean (SD)	Minimum	Maximum
Intact Uterine Horn	97.00 (15.98)	69.00	121.00
Resection			
Utero-tubal Junction—Anastomosis	23.79 (4.14)	18.00	29.50
Anastomosis—Uterine Horn	12.64 (2.39)	8.50	14.50
Total Length	36.63 (3.49)	32.00	41.00
Uterine Body	6.56 (2.09)	3.50	10.00
Cervix	18.31 (5.04)	8.50	25.00
Vagina			
Cranial	19.63 (5.01)	13.00	28.00
Caudal	12.44 (0.94)	11.00	14.00
Total Length	32.06 (5.05)	25.50	40.00
Oviduct			
Ipsilateral	28.50 (0.82)	25.50	32.00
Contralateral	27.88 (1.69)	21.00	35.50

**Table 3:** Quantitative counts of pre-implantation oocytes and embryos recovered during the post-slaughter embryo collections in the gilts (*n* = 8).

Unfertilized Oocyte	Fertilized Oocyte	2–4 Cell	4–8 Cell	Morula	Total
18	4	1	37	30	90

## Data Availability

The data that support the findings of this study are available from the corresponding authors upon reasonable request.

## Abbreviations

The following abbreviations are used in this manuscript:

CL	Corpora lutea
d	Day
h	Hour
IM	Intramuscular
IV	Intravenous
LSM	Least squares mean
P0	Parity zero
PGF2α	Prostaglandin F2alpha
SD	Standard deviation
UH	Uterine horn
UTJ	Utero-tubal junction
